# Introduction to the SIMPLE Macro, a Tool to Increase the Accessibility of 24-Hour Dietary Recall Analysis and Modeling

**DOI:** 10.1093/jn/nxaa440

**Published:** 2021-03-09

**Authors:** Hanqi Luo, Kevin W Dodd, Charles D Arnold, Reina Engle-Stone

**Affiliations:** Institute for Global Nutrition, University of California, Davis, Davis, CA, USA; Department of Nutrition, University of California, Davis, Davis, CA, USA; National Cancer Institute, NIH, Bethesda, MD, USA; Institute for Global Nutrition, University of California, Davis, Davis, CA, USA; Department of Nutrition, University of California, Davis, Davis, CA, USA; Institute for Global Nutrition, University of California, Davis, Davis, CA, USA; Department of Nutrition, University of California, Davis, Davis, CA, USA

**Keywords:** dietary analysis, dietary modeling, National Cancer Institute, micronutrients, 24-h dietary recalls

## Abstract

**Background:**

Information on long-term dietary intake is often required for research or program planning, but surveys routinely use short-term assessments such as 24-h recalls (24HRs). Methods to reduce the impact of within-person variation in 24HRs, such as the National Cancer Institute (NCI) method, typically require extensive training and skill.

**Objectives:**

We introduce the Simulating Intake of Micronutrients for Policy Learning and Engagement (SIMPLE) macro, a new tool to increase the accessibility of 24HR analysis. We explain the underlying theory behind the tool and provide examples of potential applications.

**Methods:**

The SIMPLE macro connects the core NCI statistical code to estimate usual intake distributions and includes additional code to enable advanced analyses such as predictive modeling. The related SIMPLE-Iron macro applies the full probability method to estimate inadequate iron intake, and the SIMPLE-1D macro is used for descriptive or modeling analyses of data with a single 24HR per person. The macros and associated documentations are freely available. We analyzed data from the US National Health and Nutrition Examination Survey (NHANES) and the Cameroon National Micronutrient Survey to compare the SIMPLE macro to *1*) the core NCI code using the Estimated Average Requirement cut point method, and *2*) the IMAPP software for iron only, and to demonstrate the applications of the SIMPLE macro for estimating usual intake and predictive modeling.

**Results:**

The SIMPLE macro generates identical results to the core NCI code. The SIMPLE-Iron macro also produces estimates of inadequate iron intake comparable to the IMAPP software. The examples demonstrate application of the SIMPLE macro to *1*) descriptive analyses of nutrient intake from food and supplements (NHANES), and *2*) analyses accounting for breast-milk nutrient intake and modeling fortification and supplementation programs (Cameroon).

**Conclusions:**

The SIMPLE macros may facilitate the analysis and modeling of dietary data to inform nutrition research, programs, and policy.

See corresponding article on page 1059.

## Introduction

Poor diet contributes to each of the diverse forms of malnutrition that now coexist across the globe, from wasting and micronutrient deficiencies to diet-related noncommunicable disease (1, 2). High-quality information on usual dietary intake is a critical input to plan effective and efficient programs to improve nutritional status and related outcomes (3). Such data are needed to understand food consumption patterns, assess which nutrients may be of public health concern (either because intakes are low or because they are high), and determine which population subgroups are most affected. Information on the predicted effect of hypothetical nutrition intervention programs on dietary intake and other outcomes may also guide decisions regarding how to address population-specific nutrition problems.

Analysts of dietary intake data are typically interested in information on usual, or long-term average, nutrient intake, including parameters such as the mean, median, and interquartile range (IQR) of the distribution, or the prevalence of inadequate or excessive usual nutrient intakes. In contrast, surveys routinely rely on short-term assessments of dietary intake, such as 24-h recalls (24HRs), to collect information on population intake. Estimation of usual intake is challenging when such methods are used, however, because the distribution of dietary intake measured on ≥1 d is subject to substantial within-person variation (due largely to the day-to-day variation of a person's dietary intake in relation to their usual intake). To address this problem, researchers have developed methods to mathematically adjust for this day-to-day variation to estimate the distribution of usual intake using short-term data (4–8). Among these methods, the National Cancer Institute (NCI) method has been widely used for analysis of foods or nutrients in 24HR data (8), using freely available SAS macro code. Although the NCI method is very flexible and powerful, it has a fairly steep learning curve. Applying the method requires understanding the theoretical basis for analyzing dietary intake data, mastery of multiple SAS macros, and a high proficiency in SAS coding to enable data-set-specific modifications.

Researchers and policymakers are also often interested in knowing how a population's diet can be improved by hypothetical nutrition-related interventions, such as micronutrient fortification, dietary supplementation, or interventions intended to improve diet quality or diversity (9–15). Modeling the effect of nutrition interventions involves complex dietary analysis and can be conducted with the NCI macro code, but further effort and technical skill are necessary to modify the input data or code to meet these objectives. Organizations that are willing and able to invest in building this capacity must also consider the time required to do so; this time lag may result in lost opportunities to make dietary data available during critical decision-making periods. The need for global efforts to standardize guidance for dietary data analysis and interpretation has been repeatedly emphasized by recent nutrition initiatives (3, 16, 17).

To improve the utilization of dietary data by helping researchers and analysts conquer the difficulties of applying the NCI method, the University of California, Davis (UCD) and NCI jointly developed the UCD/NCI Simulating Intake of Micronutrients for Policy Learning and Engagement (SIMPLE) macro. The SIMPLE macro provides a single tool that can be used for both basic and advanced dietary analysis and modeling. Two primary advantages of this tool are that *1*) it combines the several existing NCI macros that need to be used jointly to estimate distributions of usual intake for any dietary components consumed by nearly everyone nearly every day (“nearly-daily”), which reduces the time required for analysts to do this linking themselves; and *2*) in addition to typical descriptive analyses, the tool allows for advanced dietary analysis and modeling, such as incorporating nutrient intake from supplements and breast milk into total nutrient intake or simulating the effect of micronutrient fortification of foods. The dietary components that can be analyzed with the SIMPLE macro include nutrients, energy, food, or other dietary components, but, for simplicity, in this article we use “nutrients” to refer to all dietary components consumed nearly daily.

Variations of this macro, the SIMPLE-Iron and SIMPLE-1D, are available to address specific challenges, such as the need to apply the full probability method to assess nutrient adequacy for iron and the availability of only a single day of 24HR per person (18). The SIMPLE, SIMPLE-Iron, and SIMPLE-1D macros and example codes are available online (Open Science Framework link: https://osf.io/ytd34/); the corresponding SIMPLE macro user manual and a detailed description of the method used in the SIMPLE-Iron are provided with this article (**Supplemental User Manual 1**, **Supplemental Method 1**). We hope the detailed user manual and examples will allow users to modify the example codes for their own dietary research.

The objectives of this article are to describe the general structure and features of the SIMPLE macro, explain the underlying theory, and present example applications of the SIMPLE macro for descriptive analyses and dietary modeling. We first provide an overview of the NCI macros, which form the underlying analytical method of the SIMPLE macro. We then describe the general structure and features of the SIMPLE macro (including the SIMPLE-1D and SIMPLE-Iron variations) and compare the usual intake distribution estimated by the SIMPLE macro with that generated by the standard NCI macros; we also compare the results from applying the full probability method for estimating inadequate iron intake using the SIMPLE-Iron macro with the same analysis conducted with the IMAPP software (5). Next, we describe the core methods applied within the SIMPLE macro to implement selected advanced analyses and modeling techniques. Finally, we present example applications of the SIMPLE macro to national survey data from the United States (19) and Cameroon (20) and comment on the strengths and limitations of the SIMPLE macro with respect to other approaches to dietary analysis.

## Methods

### Overview of the NCI method for estimating usual nutrient intake

**FIGURE 1:**
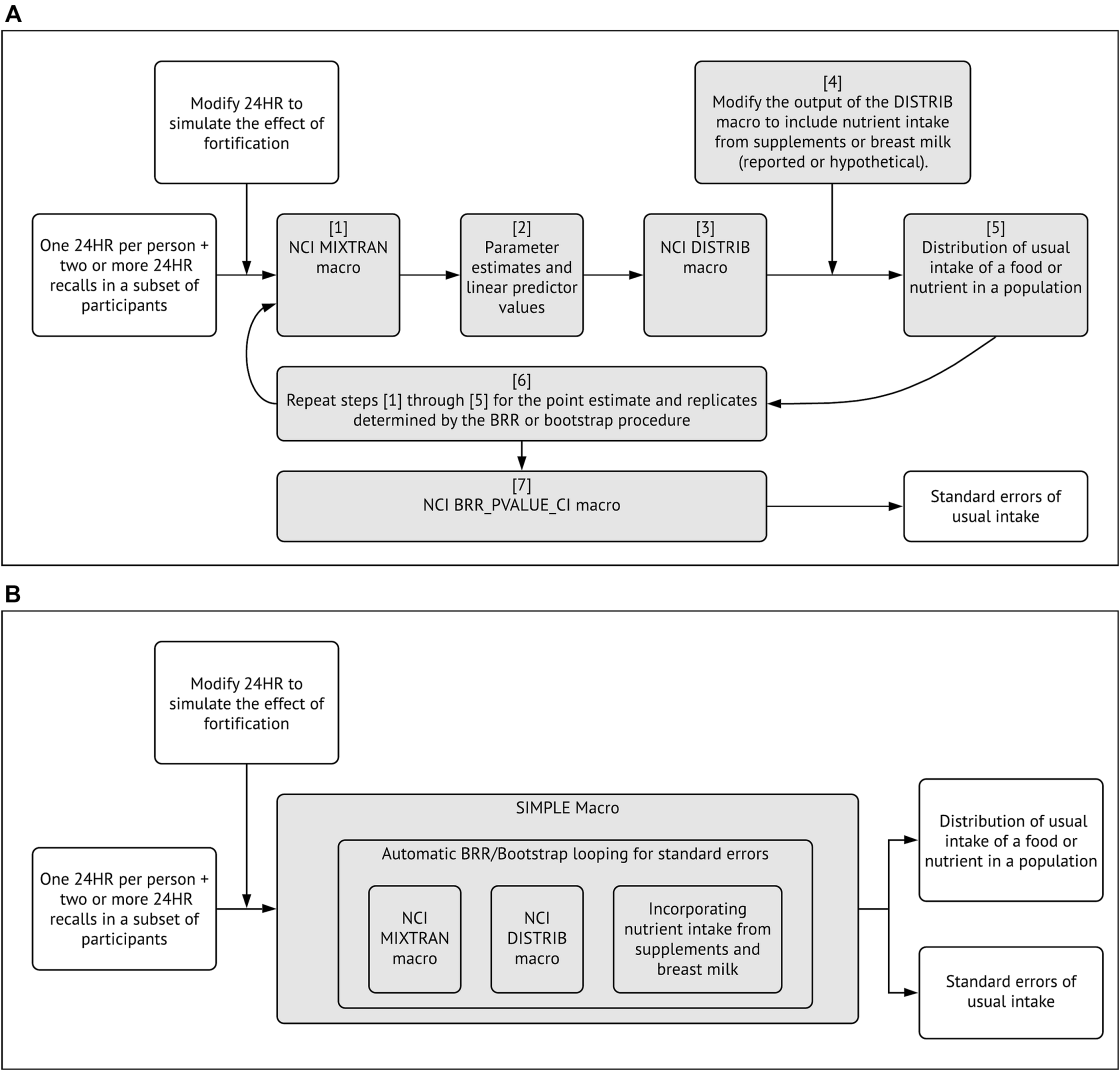
Comparison of the procedures between the SIMPLE macro and NCI method. BRR, Balanced Repeated Replication; NCI, National Cancer Institute; SE, Standard Errors; SIMPLE, Simulating Intake of Micronutrients for Policy Learning and Engagement; UCD, University of California, Davis; 24HR, 24-h recall. The shaded boxes show that the SIMPLE macro condenses steps [1] to [7] of the NCI method. For data sets with a single 24HR per person, the NCI MIXTRAN macro will be replaced with the UCD/NCI TRAN1 macro and an external variance ratio to estimate the distribution of usual intake, and the SIMPLE macro will be replaced with the SIMPLE-1D macro and an external variance ratio to estimate the distribution of usual intake.

The NCI method for estimating usual intake distributions is implemented in 2 macros written in the SAS programming language: MIXTRAN and DISTRIB (21). **Figure 1**A shows an overview of how the NCI method estimates usual intake distributions. The MIXTRAN macro transforms dietary intake data to approximate normality, models the transformed data as the sum of a covariate-based prediction function and 2 kinds of deviations from that function (between- and within-individual deviations), and produces parameter estimates and covariate-based predictions used as inputs for the DISTRIB macro. In turn, the DISTRIB macro generates a simulated data set of estimated usual intakes based on the 24HRs by, first, multiply imputing the between-person deviations and then adding them to each sampled person's prediction, and, second, converting the imputed values back to the original scale. By only imputing between-person deviations and by using a numerical integration for the back-transformation, this approach analytically corrects for within-person variation. Percentiles of usual intake and proportions of the population with usual intake below or above specified values can be estimated from the simulated data set. Advanced analyses are also supported by using the option of saving the data set. The MIXTRAN macro will not run on data sets without repeated recalls on at least a subsample of individuals, but using the approach of Luo et al. (22), the MIXTRAN macro can be replaced with a different macro (TRAN1) that uses an external ratio of within- and between-person variance and more restrictive assumptions to perform the modeling step with only one 24HR per person.

Obtaining Standard Errors (SEs) essential to inference for parameter estimates from MIXTRAN and usual intake percentiles or proportions from DISTRIB is not straightforward (Figure 1A). For some complex survey designs, SEs can be calculated using the Balanced Repeated Replication (BRR) method (23); for other survey designs or in simple randomized samples, a bootstrap approach is required. Both methods entail running the MIXTRAN/DISTRIB combination many times on modified input data, saving the desired output each time, then estimating SEs from the replicated output using code such as that provided by the NCI BRR_PVALUE_CI macro (21). A substantial amount of SAS programming is required to automate this approach for application to a variety of nutrients and/or data sets, with careful attention paid to properly combining the MIXTRAN and DISTRIB macros.

### Overview of the NCI/UCD SIMPLE macro

The SIMPLE macro is a single macro that links 3 NCI macros, the MIXTRAN, DISTRIB, and BRR_PVALUE_CI, to facilitate estimation of usual intake distributions for food and nutrients consumed “nearly-daily.” The SIMPLE macro also includes additional features, such as carrying out checks on the input data sets and supporting a variety of specific analyses, including modeling nutrition-related interventions (13, 15, 24, 25); however, before this article, the associated codes and analytical details have not been published, which may have limited the extent to which other researchers could then apply these methods to their own data.

Figure 1 shows the relation between the SIMPLE macro and the core NCI macros. Because the SIMPLE macro serves as a connector of the existing NCI macros without modifying any core part of the NCI macros, the results of using the SIMPLE macro are exactly the same as those of using several NCI macros jointly. We applied the SIMPLE macro to the example data provided by the NCI website for estimating the usual intake distribution of added sugars (21). The results generated by the SIMPLE macro were identical to the results provided on the NCI site that correspond to the example data and codes for the MIXTRAN, DISTRIB, and BRR_PVALUE_CI macros (**Table 1**). This observation has been confirmed through extensive testing of the SIMPLE macro on 3 nationally representative surveys: multiple cycles of the National Health and Nutrition Examination Survey (NHANES) (19), the Cameroon National Micronutrient Survey (CNMS) (20), and the Ethiopian National Food Consumption Survey (26).

**TABLE 1:** Usual added sugar intake estimated by the NCI MIXTRAN, DISTRIB, and BRR_PVALUE_CI macros and the UCD/NCI SIMPLE macro in males ≥4 y of age using the example data from the NCI website^1^

		Mean, g/d		Usual intake <10 g/d, %		25th percentile, g/d		50th percentile (median), g/d		75th percentile, g/d	
Subgroup	*n*	NCI	SIMPLE	NCI	SIMPLE	NCI	SIMPLE	NCI	SIMPLE	NCI	SIMPLE
Overall	6723	25.5 ± 0.4	25.5 ± 0.4	11.5 ± 1.1	11.5 ± 1.1	14.8 ± 0.4	14.9 ± 0.4	23.0 ± 0.4	23.1 ± 0.4	33.4 ± 0.6	33.5 ± 0.6
4–8 y	1010	27.9 ± 0.8	28.0 ± 0.8	6.1 ± 1.2	6.0 ± 1.2	17.7 ± 0.7	17.7 ± 0.8	25.7 ± 0.8	25.9 ± 0.9	35.7 ± 1.1	35.9 ± 1.1
9–13 y	1332	33.1 ± 1.1	33.2 ± 1.1	2.8 ± 0.6	2.8 ± 0.6	21.7 ± 0.8	21.8 ± 0.8	30.7 ± 1.0	30.9 ± 1.0	42.1 ± 1.4	42.1 ± 1.5
19–30 y	1021	30.4 ± 1.3	30.5 ± 1.3	4.3 ± 1.3	4.3 ± 1.2	19.6 ± 1.2	19.7 ± 1.2	28.2 ± 1.3	28.2 ± 1.3	38.9 ± 1.5	39.0 ± 1.5
31–50 y	1402	26.8 ± 0.7	26.8 ± 0.7	7.2 ± 1.3	7.1 ± 1.2	16.8 ± 0.7	16.9 ± 0.7	24.7 ± 0.7	24.7 ± 0.7	34.5 ± 0.9	34.5 ± 0.9
51–70 y	1176	18.1 ± 0.6	18.1 ± 0.6	23.7 ± 2.1	23.6 ± 2.0	10.3 ± 0.5	10.3 ± 0.5	16.2 ± 0.6	16.1 ± 0.6	23.8 ± 0.8	23.8 ± 0.8
>70 y	782	15.6 ± 0.4	15.7 ± 0.4	31.9 ± 2.2	32.0 ± 2.2	8.6 ± 0.4	8.6 ± 0.4	13.7 ± 0.4	13.8 ± 0.4	20.6 ± 0.5	20.7 ± 0.5

1 Values are mean ± SE. The results estimated by the NCI MIXTRAN, DISTRIB, BRR_PVALUE_CI macros method were generated by using the example codes provided by the NCI website (available from: https://prevention.cancer.gov/research-groups/biometry/measurement-error-impact/software-measurement-error/single-regularly-consumed-or-1). NCI, National Cancer Institute; SIMPLE, Simulating Intake of Micronutrients for Policy Learning and Engagement; UCD, University of California, Davis.

**FIGURE 2:**
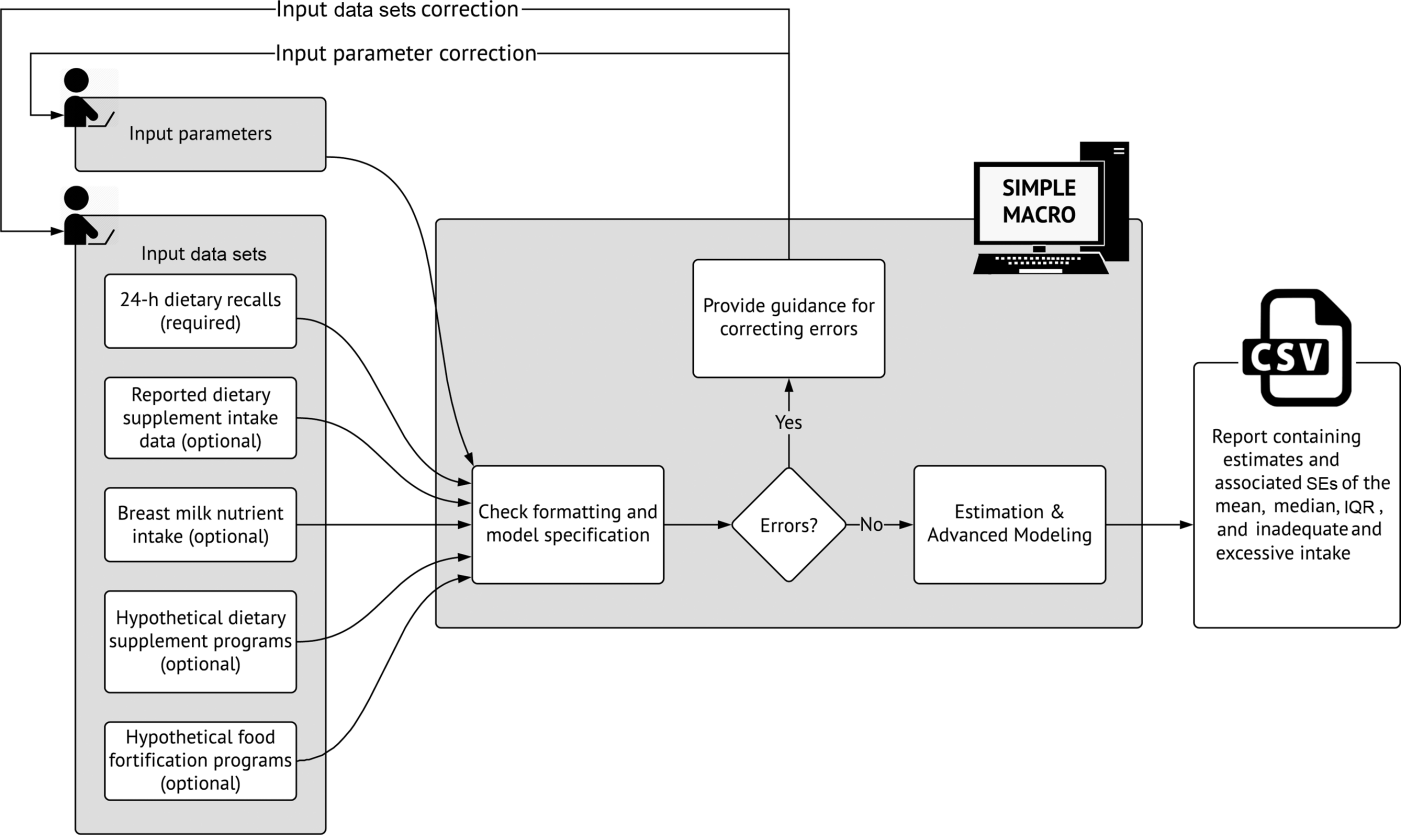
Overview of the SIMPLE macro inputs, processes, and outputs. IQR, interquartile range; SE, Standard Errors; SIMPLE, Simulating Intake of Micronutrients for Policy Learning and Engagement. Input parameters means that users should specify the relevant variable names, such as the names of nutrient intake, survey weight parameters, and unique identification as inputs to the SIMPLE macro.

**Figure 2** shows an overview of the SIMPLE macro inputs, processes, and outputs. Data sets and macro options are checked for formatting and model specification errors before starting analysis; if any errors are detected, the macro provides guidance for correcting them. The output from a successful run includes a formatted spreadsheet (suitable for use in reports or manuscripts) containing estimates and associated SEs for characteristics of the usual intake distribution, such as the mean, median, IQR, and proportions of the population with inadequate or excessive nutrient intake. The core SIMPLE macro estimates inadequate nutrient intake using the Institute of Medicine Estimated Average Requirement (EAR) cut-point method, defining prevalence of inadequate intake as the proportion of individuals with intake below each individual's age- and sex-specific EAR for a given nutrient; likewise, excessive intake is defined as intake above the corresponding Tolerable Upper Intake Level (UL) (27). The SIMPLE macro also outputs model parameters such as within- and between-person variance components and coefficients for included covariates that influence the distribution of usual intake.

**FIGURE 3:**
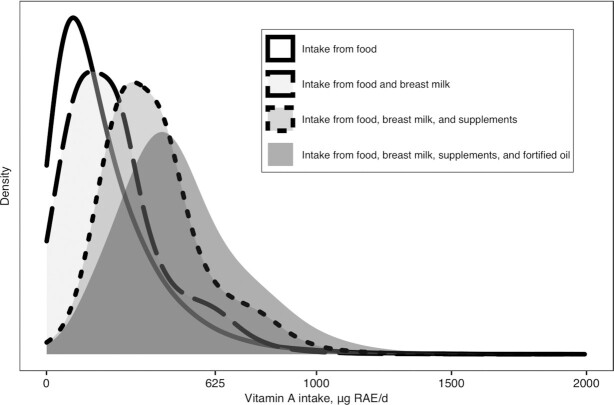
Distribution of vitamin A intake (µg RAE/d) from food, breast milk, simulated supplementation program and oil fortification program among children 1 to 5 years of age from the Cameroon National Micronutrient Survey 2009, estimated using the UCD/NCI SIMPLE macro. NCI, National Cancer Institute; RAE, Retinol Activity Equivalent; UCD, University of California, Davis. We assumed that breastfed children in the North, South, and Cities consumed 550.6, 232.2, and 473.2 µg RAE/d of vitamin A from breast milk. The regional variation in vitamin A intake from the breast milk are due to varying vitamin A concentrations in mothers’ breast milk by region. The simulated dietary supplementation program was assumed to deliver a daily dose of 167 μg retinol to a random sample of 90% of the target population. The simulated oil fortification program was assumed to fortify all industrially refined cooking oil with 12 μg retinol/g of oil.

### SIMPLE-Iron

The EAR cut-point method requires several assumptions, including the assumption that the distributions of nutrient requirements are symmetrical (27), which is known to be violated in the case of iron requirements for populations such as menstruating women. Although the SIMPLE macro can be used to estimate some parameters of iron intake distribution (mean, median, and percentiles), the SIMPLE macro, which uses the EAR cut-point method, is not appropriate for computing the prevalence of inadequate usual iron intake. To enable estimation of the prevalence of inadequate iron intake, we developed the SIMPLE-Iron macro, which is a variation of the SIMPLE macro that applies the full probability method instead of the EAR cut-point method to estimate inadequate iron intake (18). Although the SIMPLE-Iron macro was specifically developed to analyze usual iron intake, it can also be modified to apply the full probability method to other nutrients of interest. A detailed description of the methods and assumptions used in the SIMPLE-Iron macro is provided with this article (Supplemental Method 1). We compared the results of the SIMPLE-Iron macro with the prevalence of inadequate iron intake as estimated by the IMAPP software (5), which also applies the full probability method for iron (18) among children and adolescents 9–18 y of age using NHANES 2011–2014 (**Table 2**). When the SIMPLE macro is applied in a way that is analogous to the way IMAPP functions (stratifying analyses of different age and sex groups, without applying any other covariates), the results from the IMAPP software and SIMPLE macro are similar, and the estimates from IMAPP are within the CIs of the results generated by the SIMPLE macro (by default, IMAPP only produces SEs for percentiles when estimating iron usual intake; therefore, we only present the CIs for percentiles).

**TABLE 2:** Usual iron intake estimated by the IMAPP software and the UCD/NCI SIMPLE macro among children and adolescents 9–18 y of age by sex and age using data from the NHANES 2011–2014^1^

	Inadequate intake, %		Mean, mg/d		25th percentile, mg/d		50th percentile (median), mg/d		75th percentile, mg/d	
Subgroup	IMAPP	SIMPLE	IMAPP	SIMPLE	IMAPP	SIMPLE	IMAPP	SIMPLE	IMAPP	SIMPLE
Female (9–13 y)	1.2	0.6 (0, 2.4)	13.0	13.1 (12.1, 14.0)	10.7 (10.1, 11.3)	10.8 (9.7, 11.8)	12.8 (12.3, 13.3)	12.8 (12.0, 13.5)	15.2 (14.5, 15.9)	15.0 (13.2, 16.9)
Female (14–18 y)	14.5	15.2 (12.4, 18.0)	12.3	12.3 (11.8, 12.9)	9.8 (9.3, 10.3)	9.8 (9.3, 10.3)	11.9 (11.4, 12.4)	11.9 (11.4, 12.4)	14.3 (13.6, 15.0)	14.5 (13.6, 15.3)
Male (9–13 y)	0.0	0.0 (0, 0.1)	15.8	15.9 (14.5, 17.3)	13.4 (12.8, 14.0)	13.6 (12.2, 15.0)	15.5 (14.9, 16.1)	15.6 (14.3, 17.0)	18.0 (17.2, 18.8)	17.9 (16.0, 19.9)
Male (14–18 y)	4.6	2.7 (0, 5.5)	19.0	18.9 (17.5, 20.3)	13.8 (13.0, 14.6)	13.6 (12.1, 15.0)	17.6 (16.8, 18.4)	17.7 (16.3, 19.0)	22.6 (21.2, 24.0)	22.9 (21.1, 24.7)

1 Values generated from the SIMPLE macro are mean (95% CI). IMAPP only produces SEs for percentiles by default when estimating iron usual intake; therefore, we only present the CIs for percentiles. Estimates from IMAPP are within the CIs of the estimates from the SIMPLE macro. Usual iron intake was estimated based on the assumption of 18% iron absorption and mixed oral contraceptive use among females. The SIMPLE macro was applied in a way that is analogous to the way IMAPP functions (stratifying analyses of different age and sex groups, without applying any other covariates). NCI, National Cancer Institute; NHANES, National Health and Nutrition Examination Survey; SIMPLE, Simulating Intake of Micronutrients for Policy Learning and Engagement; UCD, University of California, Davis.

### SIMPLE-1D

Although it is always recommended to collect repeated 24HRs per person, some food consumption surveys and research studies have included only 1 single 24HR per person, owing to logistical challenges or limited resources (25, 28). Therefore, the SIMPLE-1D macro was developed as an extension to the SIMPLE macro and can be used to perform the same types of descriptive and advanced analyses on these “single-day” data sets. Technically, just as the SIMPLE macro leverages the ability to manipulate the *output* from the NCI DISTRIB macro, the SIMPLE-1D macro leverages the ability to manipulate the *input* to DISTRIB by replacing the MIXTRAN with the TRAN1 macro, which uses external data on the ratio of within- and between-person variance (22).

### Application of the SIMPLE macros for advanced analyses and modeling

In the following paragraphs, we explain how the SIMPLE macro modifies the NCI macros to perform dietary analysis and modeling beyond the estimation of usual intake distributions. The SIMPLE macro leverages the ability to manipulate the simulated data set produced by DISTRIB to easily perform some advanced analyses. The SIMPLE macro expands on this technique by in addition incorporating variables that add specified amounts to the simulated usual intakes themselves. This option is intended to account for sources of usual nutrient intake not routinely captured by 24HRs, such as dietary supplements and breast milk, which are often quantified via longer-term assessments (29, 30). For dietary supplements in particular, these values may cluster around a small number of dosages that are extremely high compared with the continuous distribution of usual intakes from food sources. For example, iron supplements commonly provide 30 mg Fe/d, which is likely to be substantially higher than usual food iron intake. In the United States, >50% of the adult population regularly consumes dietary supplements, and dietary supplements substantially contribute to total nutrient intake (31, 32). Thus, when evaluating the prevalence of inadequate or excessive intake, estimating usual nutrient intake from food sources only provides an incomplete picture of dietary patterns (29). Similarly, for infants and young children, breast milk is an important source of nutrients. Whereas individual-level data on usual breast milk intake and nutrient composition are rarely available, published estimates can be used to approximate the contribution of breast milk to total nutrient intake. To capture nutrient intake from dietary supplements (reported or hypothetical) or breast milk intake, the SIMPLE macro adopts the “shrink-then-add” (30) approach to estimate distributions of usual intake from multiple data sources with different temporal scopes. The macro can use either individual-level data (e.g., from a dietary supplement questionnaire or data on an individual child's breast milk intake measured using isotope dilution) or group-level data (e.g., estimated average nutrient intake from breast milk across age groups or geographic strata). The latter case is also relevant for simulating the effects of national dietary supplement distribution programs, where a fixed dosage of a dietary supplement is proposed (e.g., 30 mg/d of iron supplements for pregnant women) (33) and coverage of dietary supplements is hypothesized or estimated from external data sources (14, 15, 25).

Like supplementation, fortification of staple foods, condiments, or other products can be an effective means to reduce micronutrient deficiency. Modeling is useful to assess the likely impact of such a strategy before investing in a national program (14, 15, 25). Because fortification involves changes to the nutrient content of foods which are captured by a 24HR, it is appropriate to modify 24HR observations to reflect hypothetical fortification before applying the NCI method (i.e., the “add-then-shrink” method) (30). This modeling can be accomplished by adjusting the food composition table value for the hypothetical fortified product, or in the 24HR database by multiplying the amount of fortifiable food consumption by the fortification level and adding this to the total nutrient intake calculated without fortification.

In all, the extended advanced modeling capabilities of the SIMPLE macro allow evaluation of the effects of proposed combinations of fortification and supplementation programs on inadequate or excessive nutrient intake. Researchers and policy advocates can use this information to answer questions relevant to program or policy design, such as comparing the effectiveness and safety of alternative fortification scenarios or supplement distribution strategies.

## Results



In this section, we present 4 example analyses for the purpose of illustrating the potential applications of the SIMPLE macro. The code, sample data, and a detailed user guide are available online (Open Science Framework: https://osf.io/ytd34/). Users can download additional results from all the examples online (https://osf.io/8ygh3/download). The first example uses data from adult women in the NHANES 2011–2014 (19) to perform a descriptive analysis of usual calcium intake from food sources alone and from the combination of food sources and dietary supplements. The second example uses the same data from the NHANES to estimate inadequate iron intake using the full probability method among children and adolescents 9–18 y old with the SIMPLE-Iron macro. The third example uses data from the CNMS (20) to model the effects of hypothetical vitamin A fortification and supplementation programs among preschool children, accounting for the contribution of breast milk intake to vitamin A intake. The fourth uses the first 24HR per person from the CNMS and implements the same analyses as the third example, to illustrate use of the SIMPLE-1D macro. We strongly recommend that users thoroughly review the NCI measurement error webinar series (34) and the measurement error webpage (35) that explains the theory underpinning the SIMPLE macro. We also recommend that users personally work through the provided examples to gain practical experience before applying the SIMPLE macro to their own studies.

### Example 1: Descriptive analysis using the EAR cut-point method and data from NHANES 2011–2014

This example uses data on women 19 y of age and older from NHANES 2011–2014 (19). In this population, individual women have different corresponding EAR and UL values owing to their physiological status, such as pregnancy or lactation. In this example, we applied the SIMPLE macro to estimate *1*) the distribution of usual calcium intake from food sources and the proportions of the population with inadequate and excessive usual intakes using a single cutoff for all individuals; *2*) the distribution of usual calcium intake and prevalence of inadequate and excessive intakes from food sources using individual-specific EAR and UL cutoffs (i.e., applying appropriate cutoffs to account for the age of each individual in the data set); and *3*) the distribution of usual calcium intake and prevalence of inadequate and excessive intakes accounting for both food sources and dietary supplements, and using individual-specific EAR and UL cutoffs. The BRR method was used to estimate SEs for these results. The results for Example 1 are presented in **Table 3**, which illustrates the direct output of the SIMPLE macro (although formatted to adhere to journal policy). Our analysis shows that whereas the mean and percentiles of total nutrient intake were the same in the first 2 analyses, the analysis using individual-specific EAR cutoffs estimated a substantially higher prevalence of inadequate calcium intake among adult women (57.1%) than the analysis using a single EAR value (45.8%). After including calcium intake from supplements, the mean calcium intake was estimated to increase from 862 mg/d to 1071 mg/d, the inadequate calcium intake to decrease from 57.1% to 40.1%, and the excessive intake to increase from 0% to 4.2%. Therefore, applying individual-specific EAR and UL values may change the prevalence of inadequate or excessive nutrient intakes (theoretically increasing the accuracy of the analysis), and including nutrient intake from supplements to capture all sources of the nutrient can change the usual nutrient distribution and prevalence of inadequacy.

**TABLE 3:** Usual calcium intake from food and/or supplements among women 19 y of age and older by race and ethnicity using data from the NHANES 2011–2014, estimated using the UCD/NCI SIMPLE macro^1^

Race	*n*	Inadequate intake, %	Excessive intake, %	Total nutrient intake, mg/d	Nutrient intake from supplements, mg/d	25th percentile of total nutrient intake, mg/d	50th percentile (median) of total nutrient intake, mg/d	75th percentile of total nutrient intake, mg/d
Calcium intake from food using single EAR (800 mg/d) and UL (2500 mg/d)								
Overall	4110	45.8 ± 1.5	0.0 ± 0.0	862 ± 12	0 ± 0	656 ± 10	830 ± 11	1033 ± 16
Mexican American	442	36.6 ± 2.9	0.0 ± 0.0	927 ± 20	0 ± 0	714 ± 22	895 ± 20	1103 ± 22
Other Hispanic	397	50.8 ± 3.6	0.0 ± 0.0	824 ± 24	0 ± 0	629 ± 22	795 ± 24	984 ± 27
Non-Hispanic white	1659	41.1 ± 1.9	0.0 ± 0.0	894 ± 15	0 ± 0	688 ± 12	861 ± 14	1064 ± 20
Non-Hispanic black	1024	63.6 ± 2.0	0.0 ± 0.0	742 ± 13	0 ± 0	562 ± 13	712 ± 13	891 ± 15
Non-Hispanic Asian	471	62.9 ± 3.3	0.0 ± 0.0	747 ± 21	0 ± 0	563 ± 19	715 ± 21	895 ± 25
Other race—including multiracial	117	62.4 ± 7.8	0.0 ± 0.0	751 ± 52	0 ± 0	565 ± 45	718 ± 51	904 ± 60
Calcium intake from food using individual-specific EARs and ULs								
Overall	4110	57.1 ± 1.4	0.0 ± 0.0	862 ± 12	0 ± 0	656 ± 10	830 ± 11	1033 ± 16
Mexican American	442	42.5 ± 2.6	0.0 ± 0.0	927 ± 20	0 ± 0	714 ± 22	895 ± 20	1103 ± 22
Other Hispanic	397	59.8 ± 2.8	0.0 ± 0.0	824 ± 24	0 ± 0	629 ± 22	795 ± 24	984 ± 27
Non-Hispanic white	1659	54.4 ± 1.8	0.1 ± 0.0	894 ± 15	0 ± 0	688 ± 12	861 ± 14	1064 ± 20
Non-Hispanic black	1024	71.5 ± 1.8	0.0 ± 0.0	742 ± 13	0 ± 0	562 ± 13	712 ± 13	891 ± 15
Non-Hispanic Asian	471	70.7 ± 2.9	0.0 ± 0.0	747 ± 21	0 ± 0	563 ± 19	715 ± 21	895 ± 25
Other race—including multiracial	117	68.2 ± 7.8	0.0 ± 0.0	751 ± 52	0 ± 0	565 ± 45	718 ± 51	904 ± 60
Calcium intake from food and supplements using individual-specific EARs and ULs								
Overall	4110	40.1 ± 1.2	4.2 ± 0.4	1071 ± 14	209 ± 1	733 ± 11	973 ± 13	1301 ± 21
Mexican American	442	35.3 ± 2.5	1.0 ± 0.3	1017 ± 24	90 ± 1	755 ± 22	962 ± 22	1217 ± 29
Other Hispanic	397	49.1 ± 2.8	2.5 ± 0.9	953 ± 34	129 ± 2	668 ± 22	865 ± 26	1123 ± 39
Non-Hispanic white	1659	34.7 ± 1.4	5.5 ± 0.6	1146 ± 18	252 ± 1	790 ± 13	1045 ± 17	1392 ± 27
Non-Hispanic black	1024	60.1 ± 2.0	1.1 ± 0.2	851 ± 17	109 ± 1	597 ± 14	779 ± 15	1019 ± 21
Non-Hispanic Asian	471	49.8 ± 2.8	2.5 ± 0.7	960 ± 26	215 ± 2	638 ± 21	859 ± 28	1180 ± 35
Other race—including multiracial	117	55.6 ± 8.0	2.6 ± 1.3	901 ± 70	149 ± 4	602 ± 51	794 ± 63	1055 ± 84

1 Values are mean ± SE. Individual-specific EARs refers to EAR = 800 mg/d for women aged ≤50 y and EAR = 1000 mg/d for women aged >50 y; individual-specific ULs refers to UL = 2500 mg/d for women aged ≤50 y and UL = 2000 mg/d for women aged >50 y. Table reformatted from the output of the SIMPLE macro. EAR, Estimated Average Requirement; NCI, National Cancer Institute; NHANES, National Health and Nutrition Examination Survey; SIMPLE, Simulating Intake of Micronutrients for Policy Learning and Engagement; UCD, University of California, Davis; UL, Tolerable Upper Intake Level.

### Example 2: Descriptive analysis using the full probability approach and data from NHANES 2011–2014

In this example, we used data on children and adolescents 9–18 y old from NHANES 2011–2014 (19) to demonstrate estimation of the distribution of usual iron intakes and the prevalence of inadequate iron intake using the SIMPLE-Iron macro. The SIMPLE-Iron macro applies the full probability method using age- and sex-appropriate reference values to estimate inadequate intake among children and adolescents, for whom the distribution of iron requirements has been defined by the US Institute of Medicine (IOM) Panel on Micronutrients in 2001. The default iron absorption value in the SIMPLE-Iron macro is 18%, consistent with the estimate of fractional iron absorption that was assumed by the IOM for a population with mixed diets (vegetarian and nonvegetarian) in calculating the recommended dietary iron intakes. However, the SIMPLE-Iron macro also allows users to input their own estimate of iron absorption, including the use of algorithms to estimate “absorbable iron” (Supplemental Method 1). **Table 4** presents the results of Example 2.

**TABLE 4:** Usual iron intake from food and/or supplements in children and adolescents 9–18 y of age and older by sex using data from the NHANES 2011–2014, estimated using the UCD/NCI SIMPLE macro^1^

Sex	*n*	Inadequate intake, %	Total nutrient intake, mg/d	Nutrient intake from supplements, mg/d	25th percentile of total nutrient intake, mg/d	50th percentile (median) of total nutrient intake, mg/d	75th percentile of total nutrient intake, mg/d
Iron intake from food using the full probability method							
Overall	2552	4.0 ± 0.7	15.1 ± 0.3	0.0 ± 0.0	11.5 ± 0.2	14.4 ± 0.2	17.9 ± 0.5
Male	1285	0.9 ± 0.4	17.0 ± 0.4	0.0 ± 0.0	13.5 ± 0.2	16.4 ± 0.3	19.9 ± 0.7
Female	1267	7.1 ± 1.1	13.2 ± 0.3	0.0 ± 0.0	10.3 ± 0.3	12.6 ± 0.2	15.5 ± 0.4
Iron intake from food and supplements using the full probability method							
Overall	2552	3.8 ± 0.7	16.1 ± 0.3	1.0 ± 0.01	11.7 ± 0.2	14.8 ± 0.2	18.8 ± 0.6
Male	1285	0.8 ± 0.4	17.9 ± 0.4	0.9 ± 0.02	13.6 ± 0.3	16.8 ± 0.3	20.7 ± 0.7
Female	1267	6.8 ± 1.1	14.3 ± 0.3	1.2 ± 0.02	10.4 ± 0.3	12.9 ± 0.2	16.1 ± 0.5

1 Values are mean ± SE. Table reformatted from the output of the SIMPLE macro. NCI, National Cancer Institute; NHANES, National Health and Nutrition Examination Survey; SIMPLE, Simulating Intake of Micronutrients for Policy Learning and Engagement; UCD, University of California, Davis.

### Example 3: Descriptive analysis and modeling of supplementation and fortification programs using the CNMS

In this example, we analyzed 24HRs from children 1–5 y of age from the CNMS (15, 20) to demonstrate use of the SIMPLE macro to estimate *1*) the distribution of usual vitamin A intake from food sources and the associated prevalence of inadequate vitamin A intake using age-specific EAR cutoffs (i.e., for children 1–3 y of age compared with 4–5 y of age); *2*) the distribution of usual vitamin A intake and associated prevalence of inadequacy accounting for both food sources and estimated breast milk consumption (among children reported to be breastfeeding at the time of the survey); *3*) the distribution of usual vitamin A intake and associated prevalence of inadequacy accounting for food sources, estimated breast milk consumption, and a simulated dietary supplementation program (assumed to deliver a daily dose of vitamin A containing 167 μg retinol to a random sample of 90% of the target population); and *4*) the distribution of usual vitamin A intake and associated prevalence of inadequacy accounting for food sources, estimated breast milk consumption, a simulated dietary supplementation program, and a simulated oil fortification program (assumed to fortify all industrially refined cooking oil with 12 μg retinol/g of oil). The BRR method is used to estimate SEs for these results. The results of Example 3 show the predicted increase in total vitamin A intake among children after including vitamin A intake from estimated breast milk consumption and simulated vitamin A supplementation and fortification programs (**Table 5**). **Figure 3** shows the predicted change in shape of the distribution of vitamin A intake when vitamin A intake from breast milk and dietary supplements is included, due to the relatively high amounts of vitamin A in these sources compared with the vitamin A intake from food.

**TABLE 5:** Vitamin A intake from food, breast milk, a simulated dietary supplementation program, and a simulated oil fortification program in children <5 y of age by zone using data from the Cameroon National Micronutrient Survey 2009, estimated using the UCD/NCI SIMPLE macro^1^

Intake, by zone	*n*	Inadequate intake, %	Total nutrient intake, μg RAE/d	Nutrient intake from supplements, μg RAE/d	25th percentile of total nutrient intake, μg RAE/d	50th percentile (median) of total nutrient intake, μg RAE/d	75th percentile of total nutrient intake, μg RAE/d
Vitamin A intake from food							
Overall	872	64.2 ± 6.5	212 ± 7	0 ± 0	77 ± 7	157 ± 9	288 ± 9
South	301	44.2 ± 11.7	290 ± 12	0 ± 0	141 ± 16	240 ± 15	385 ± 15
North	295	92.7 ± 4.9	101 ± 5	0 ± 0	48 ± 5	80 ± 5	130 ± 6
Cities	276	61.2 ± 8.1	214 ± 8	0 ± 0	95 ± 12	175 ± 12	292 ± 11
Vitamin A intake from food and breast milk^2^							
Overall	872	50.2 ± 2.2	254 ± 6	0 ± 0	119 ± 12	222 ± 11	329 ± 9
South	301	33.9 ± 4.8	333 ± 11	0 ± 0	185 ± 22	289 ± 16	452 ± 23
North	295	68.2 ± 2.2	161 ± 5	0 ± 0	81 ± 8	136 ± 7	251 ± 3
Cities	276	56.1 ± 2.8	227 ± 7	0 ± 0	119 ± 17	197 ± 13	308 ± 11
Vitamin A intake from food, breast milk, and a simulated dietary supplementation program^3^							
Overall	872	11.8 ± 2.2	404 ± 6	150 ± 0	270 ± 11	373 ± 11	487 ± 8
South	301	5.8 ± 1.7	482 ± 11	150 ± 0	335 ± 21	445 ± 16	605 ± 20
North	295	18.8 ± 3.0	312 ± 5	150 ± 0	239 ± 8	290 ± 7	412 ± 5
Cities	276	13.4 ± 2.2	378 ± 7	151 ± 0	269 ± 16	353 ± 13	465 ± 11
Vitamin A intake from food, breast milk, a simulated dietary supplementation program, and a simulated oil fortification program^4^							
Overall	872	6.1 ± 1.0	499 ± 8	150 ± 0	341 ± 14	462 ± 10	620 ± 12
South	301	4.3 ± 1.0	541 ± 13	150 ± 0	374 ± 20	506 ± 15	691 ± 23
North	295	7.8 ± 1.5	400 ± 8	150 ± 0	299 ± 12	396 ± 10	480 ± 8
Cities	276	7.3 ± 0.7	563 ± 12	150 ± 0	384 ± 24	537 ± 17	726 ± 16

1 Values are mean ± SE. Table reformatted from the output of the SIMPLE macro. NCI, National Cancer Institute; RAE, retinol activity equivalent; SIMPLE, Simulating Intake of Micronutrients for Policy Learning and Engagement; UCD, University of California, Davis.

2 We assumed that breastfed children in the North, South, and Cities consumed 550.6, 232.2, and 473.2 μg RAE/d of vitamin A from breast milk, respectively. The regional variation in vitamin A intake from breast milk reflects differing estimates of breast-milk vitamin A concentrations by region.

3 The simulated dietary supplementation program was assumed to deliver a daily dose of 167 μg retinol to a random sample of 90% of the target population.

4 The simulated oil fortification program was assumed to fortify all industrially refined cooking oil with 12 μg retinol/g of oil.

### Example 4: Descriptive analysis and modeling of supplementation and fortification programs using the CNMS with only a single 24HR per person

To demonstrate application of the SIMPLE-1D macro, we used the same data set as the previous example (20) but retained only the first 24HR per person. The example includes all 4 scenarios of vitamin A analysis and modeling among children 1–5 y of age as shown in Example 3. For single-day dietary data, users cannot estimate the ratio of within- and between-person variance components internally (i.e., from the same data set), which they can when they analyze dietary data that include repeated recalls on the same person. Thus, an external variance ratio, defined as a ratio estimated from a similar population from a different study, is often used (22, 36). In this example, we used the ratio of within- and between-person variance components of vitamin A intake estimated from Example 3 (i.e., an internal variance components ratio from the same data set) and, therefore, our results for Example 4 are similar to those of Example 3 (Table 5). However, if the assumed external variance ratio deviates from the true variance ratio, the resulting usual intake estimates can be biased (22, 36); therefore, users should be cautious when applying an external variance ratio to their single-day dietary data and should always conduct sensitivity analyses with a range of external ratios.

## Discussion

The UCD/NCI SIMPLE macro provides a streamlined structure for 24HR analysis that builds on the existing core NCI macros for estimating usual or long-term dietary intakes from multiple 24HRs. As for other similar dietary analysis tools, users need to understand the theoretical basis for analysis of 24HR data and have familiarity with basic computer code. With this background, the new, freely available tool simplifies both basic descriptive analyses and complex analyses of 24HR data, including accounting for supplement and breast milk intake, and modeling the impact of hypothetical fortification or supplementation programs, and their combinations. Extensions of the tool permit analyses of special cases when the full probability method is required to assess dietary adequacy, and when only a single day of dietary data is available for each person. With these in place, the tool allows “shortcuts” in programming capacity that can dramatically shorten the time from data cleaning to presentation of a final results table.

To strengthen data and information systems for nutrition and provide timely information for policy decision making, the Global Nutrition Report in 2017 proposed the concept of the “nutrition data value chain,” which has 5 critical processes—data prioritization, creation and collection, curation, analysis, and interpretation/recommendation—before the final step, decision making (16, 37). To date, enormous effort has been spent overcoming the early challenges in this chain: dietary data collection and curation. Open-source and standardized dietary data collection tools, such as ASA24 (38) and Intake24 (39), have been developed, validated, and used in multiple national surveys and research studies. For low-income countries, standardized data collection methods were proposed >2 decades ago (40). More recently, mobile- or tablet-based dietary data collection tools have been independently developed by nutrition researchers, such as the mobile application– and web-based dietary database by the International Dietary Data Expansion (INDDEX) (3) and the mobile-based, open-source dietary collection tool by Caswell et al. (41). In contrast, less progress has been achieved in streamlining the *analysis* of detailed dietary intake data, such as those collected using 24HR methods (17). Although technical guidelines exist for best practices in analysis of usual nutrient intake (34, 42), 24HR analysis still relies primarily on certain statistical packages that require both a high level of proficiency in computer coding and a deep understanding of the theoretical bases of 24HR analysis (6, 8) or software that is accessible to new users but has more limited features (5, 7).

The SIMPLE macro is a multifunctional dietary software tool that can be applied to diverse global contexts to help fill the gap in the nutrition data value chain between data collection and interpretation for decision making. Because the SIMPLE macro has simplified procedures for data processing using the core NCI method as well as the ability to automatically output a spreadsheet containing the information typically requested on usual intake (e.g., mean, median, inadequate or excessive intake), timely reporting of results can be achieved more easily. It is important to note that, because the SIMPLE macro uses the existing NCI macros, the SIMPLE macro does not shorten the computation time. Instead, the SIMPLE macro reduces the time spent on *1*) learning the in-depth theory of advanced dietary analysis and the NCI approach in particular, to allow appropriate manipulation of the NCI macros; and *2*) creating the code to modify several NCI macros and the output data sets for each NCI macro. Using the existing NCI macros for descriptive analyses requires implementing a minimum of 6 steps to apply the MIXTRAN, DISTRIB, and NCI BRR_PVALUE_CI macros, and additional steps may be required to conduct any advanced modeling work. In contrast, the SIMPLE macro serves as an “all-in-one” macro “shell” that can be used to carry out a wide range of analyses in a single step. The capability to model nutrition intervention programs, such as food fortification programs, is important to assess the likely impacts of such programs on dietary adequacy or excess; this information is useful both when generated in advance to guide the planning process, and after programs are in place, to guide later program management decisions. In the United States, scientists and policymakers rely heavily on the scientific evidence generated from analyzing NHANES (43) to develop the Dietary Guidelines for Americans. Our extensive testing demonstrates that the SIMPLE macro can be successfully applied to NHANES data, so researchers could use the SIMPLE macro to explore new research questions or to make the results of the latest cycles of NHANES available in a speedy manner. In addition, there is an increasing push to improve research capacity at institutions in low- and middle-income countries. The SIMPLE macro can facilitate these efforts by providing a user-friendly platform for researchers to conduct advanced dietary analysis and modeling, as well as report relevant findings to local governments and the international community, with substantial reductions in the time and cost required to do so.

The SIMPLE macro also has several limitations. First, the SIMPLE macro was developed to analyze “nearly-daily” consumed dietary components. The tool was designed to estimate population distributions of usual intake and does not incorporate the NCI method's approach to predict the relation between usual dietary intake and health outcomes (e.g., the relation between whole wheat consumption and cardiovascular health). Second, the tool cannot analyze dietary components that are episodically consumed by a population (e.g., whole wheat products in the United States, or preformed retinol intakes in some populations with very low animal-sourced food consumption). Third, the SIMPLE macro can only generate unbiased results when the dietary data are of high quality and represent the population's dietary intake. It cannot correct for errors in data collection or misreporting, such as extreme or unreasonable values of food or nutrient intake, that can exist in 24HR data. Careful data cleaning and mobile- or tablet-based dietary data collection applications that have the built-in feature of automatically cleaning the dietary data (e.g., range checks on portion sizes to avoid entering improbable values), in addition to rigorous, standardized training of interviewers, may help to minimize misreporting of dietary intake. Fourth, the SIMPLE macro has specific technical requirements. Users need to have both SAS (SAS Institute) and Microsoft Excel installed. As the non-academic-based SAS license is expensive, resource-constrained analysts or those who are not affiliated with an academic institution may need to partner with universities to carry out dietary analysis with this tool. In addition, users are required to have basic SAS programming skills, which requires time and access to appropriate training materials. These user requirements may block the usage of the SIMPLE macro from a wider audience. In the future, we aim to improve the use of the SIMPLE macro and address these limitations. To facilitate use of the current macro version, we have designed training materials to enhance the usage of the SIMPLE macro and build the capacity of researchers and policy analysts (training materials are available upon request). Plans to expand the current SIMPLE macro tool include the addition of new functionalities, such as analysis of episodically consumed nutrients and foods. In the longer term, we envision development of a web-based tool based on the SIMPLE macro to further increase the accessibility of the method and decrease the time and resources required to make dietary data results available.

In conclusion, although the value of dietary analysis for nutrition research and program planning is widely recognized (3), such data may be underutilized owing to the complexity of appropriate statistical analyses and the corresponding capacity needs. By providing a more user-friendly structure around the existing core NCI macros, the SIMPLE macro streamlines basic descriptive analyses of 24HR data while also providing extra functionality to enable more complex analyses. Specifically, the tool permits users to estimate the observed contributions of supplements or breast milk to usual nutrient intakes and model the effects of hypothetical nutrition intervention programs, such as supplementation or food fortification. Variations of the tool help to overcome other technical challenges, such as applying the full probability method to estimate inadequate iron intake and estimating usual intake distributions with only a single day of data per person. The SIMPLE macro has the potential to make analysis of 24HR data more accessible by reducing the time and resources needed to conduct high-quality dietary analyses, facilitating the availability of dietary intake data for research and to inform nutrition policies and programs.

## Supplementary Material

nxaa440_Supplemental_File

## Notes

Supported by Bill & Melinda Gates Foundation award #OPP1170661 (to RE-S), USDA National Institute of Food and Agriculture Hatch project 1013897 (to RE-S), and the National Cancer Institute (to KWD).

Author disclosures: the authors report no conflicts of interest.

Supplemental User Manual 1 and Supplemental Method 1 are available from the “Supplementary data” link in the online posting of the article and from the same link in the online table of contents at https://academic.oup.com/jn/.

Abbreviations used: BRR, Balanced Repeated Replication; CNMS, Cameroon National Micronutrient Survey; EAR, Estimated Average Requirement; IOM, Institute of Medicine; IQR, interquartile range; NCI, National Cancer Institute; NHANES, National Health and Nutrition Examination Survey; SE, Standard Errors; SIMPLE, Simulating Intake of Micronutrients for Policy Learning and Engagement; UCD, University of California, Davis; UL, Tolerable Upper Intake Level; 24HR, 24-h recall.
