# Supplementary material for: Introduction to the SIMPLE Macro, a Tool to Increase the Accessibility of 24-Hour Dietary Recall Analysis and Modeling
Source: J Nutr. 2021 Mar 9;151(5):1329–40. doi: 10.1093/jn/nxaa440 (PMC8112768; doi:10.1093/jn/nxaa440)
Supplement: nxaa440_Supplemental_File [file nxaa440_supplemental_file.docx]

**Online Supplemental Method 1**

**Method to estimate inadequate iron intake in the SIMPLE-Iron macro using the full probability method**

The SIMPLE-Iron macro uses the method proposed by the US Institute of Medicine (IOM) to estimate inadequate iron intake (1). This section describes the methods used in the SIMPLE-Iron macro, with focus on the two main hurdles in estimating inadequate iron intake: accounting for factors affecting iron absorption and implementing the full probability method. Although the SIMPLE-Iron macro was developed with a focus on iron, the tool can also be applied to estimate inadequate intake of other nutrients using the full probability method, if appropriate values for the distribution of nutrient requirements are substituted.

Iron absorption depends on the form of iron consumed (heme or non-heme) and other dietary components consumed with iron in the same meal (2), as well as individual iron status, and consequently iron absorption varies across populations and individuals. Options available to analysts include 1) comparing observed total iron intake directly to the reference values for dietary intake of iron (with the implicit assumption that iron absorption in the population studied is the same as that used to derive the reference intakes), 2) applying a fixed level of iron absorption selected based on population characteristics and diet (for example, assuming that iron absorption for a population consuming a vegetarian diet is relatively low at 5% or 10%; logistically, this can be done by adjusting either the dietary data or the reference values), or 3) applying an algorithm to estimate iron absorption of individuals in the population based on dietary components and/or individual iron status (3) before applying the National Cancer Institute (NCI) method to adjust within-person variation (4).

Another complexity of working with iron is that the Estimated Average Requirement (EAR) cut point method is not appropriate because the distribution of iron requirements is known to be skewed rather than symmetrical (5). To correctly estimate inadequate iron intake, researchers need to apply the full probability method. Additionally, iron requirements differ by age, gender, physiological status (non-pregnancy or non-lactation [NPNL], pregnancy, or lactation), and oral contraceptive (OC) use among women, which leads to further complexity in applying the full probability method. The SIMPLE-Iron macro applies the full probability method to estimate inadequate intake among children, teenagers, and NPNL, for whom the distribution of iron requirements has been defined by the US Institute of Medicine (IOM) (1). Currently, the SIMPLE-iron macro cannot be applied to estimate inadequate iron intake among pregnant or lactating women, for whom the distribution of iron requirements has not been characterized. We will update the SIMPLE-iron macro once estimated distributions of iron requirement for pregnant and lactating women are available.

In contrast, to estimate excessive iron intake, the cut point method is appropriate to estimate iron intake exceeding the tolerable upper intake level (UL) (6); therefore, users should apply the SIMPLE macro instead of the SIMPLE-iron macro to total iron intake from 24HR in this case to estimate excessive iron intake.

In the following section, we first describe the process used to account for iron absorption in the SIMPLE macro. Then, we describe the method to estimate inadequate iron intake for each of three groups: children (male and female 0.5 - 8 y), women (female ≥ 9 y), and men (male ≥ 9 y).

**Iron absorption**

The default level of fractional iron absorption in the SIMPLE-Iron macro is 18%, as proposed by the IOM. However, studies have shown that actual iron absorption in high-, middle-, and low- income countries is lower than 18% (7,8). Absorption is especially low (only 5% - 15%) among populations with vegetarian or high- phytate diets (2,7). In the SIMPLE-iron macro, users have the flexibility to enter their assumed level of fractional iron absorption (from 1% to 100%).

Some users may wish to apply an iron absorption algorithm (3) to total iron intake in the 24 dietary recalls (24HRs) before applying the SIMPLE-Iron macro. The output of such algorithms thus reflects “absorbable iron”, and thus users of the SIMPLE-Iron macro are assessing the usual intake of “absorbable iron” rather than the usual intake of total iron. In this case, users should enter the estimated iron absorption as 100% to avoid double correction for absorption.

**Full probability method**

First proposed by Beaton in 1974, the full probability method is used to compare the two distributions: nutrient requirements and nutrient intakes (9,10). The full probability method assigns a probability of inadequacy for each value of usual intake. The probability of inadequacy is lower for those with high intake and higher for those with low intake. The arithmetic mean (or survey-weighted arithmetic mean if the data were collected using a complex survey design) of these inadequacy probabilities is the estimated population prevalence of inadequacy. The SIMPLE-Iron macro estimates the prevalence of inadequate iron intake using the following procedure: 1) apply the NCI method to 24HR data to produce a model for usual iron intake; 2) use the model to generate a dataset containing simulated usual iron intake for a population-representative sample of “pseudo-individuals”; 3) select population-appropriate look-up table(s) for probabilities of inadequacy assigned to ranges of usual iron intake that account for differences in requirements based on age, gender, and OC use (**Supplemental table 1-3**); 4) assign the probability of inadequacy for the simulated intakes using the look-up table, accounting for any user-specified iron absorption percentages that differ from the IOM default; and finally 5) compute the average (or survey weighted average if the data were collected using a complex survey design) of the assigned probabilities to obtain the population-level prevalence of inadequate iron intake.

If the user specifies a non-default absorption percentage, the probability of inadequacy is obtained from the table entry corresponding to an adjusted iron intake obtained by multiplying the simulated usual iron intake by the user-specified percentage and then dividing by 18%. For example, if iron intake for an 8-month-old is 5.0 mg, the probability of inadequacy from the default table (**Supplemental table 1**) is 0.85. However, if the user specifies an absorption percentage of 12% then the probability of inadequacy is 0.96, corresponding to the intake range of 3.01-3.63 because the adjusted iron intake would be calculated as 5.0 × (12%/18%) = 3.33. The adjusted value is only used in the lookup of inadequacy probabilities, all other reported characteristics of the distribution are calculated from the original simulated usual intakes.

1. **Children (male and female 6 mos - 8 y)**

Children’s iron requirements differ by age (6 - 11.9 mos, 1 – 3 y, and 4 - 8 y). The SIMPLE macro applies the full probability method in estimating inadequate iron intake using age-appropriate reference values (**Supplemental table 1**).

1. **NPNL Women (female ≥ 9 y)**

Women’s iron requirements differ by age categories, indicating different menstruation status (9-13, 14-18, 19 - 50, and > 50 y^[[1]](#footnote-1)^), OC use (non-OC, OC users, and mixed population), and physiological status (NPNL, pregnancy, or lactation) (**Supplemental figure 1)**. **Supplemental table 2** provides the appropriate iron requirement values for NPNL women. Currently, the SIMPLE-Iron macro is not applicable to pregnant or lactating women, as their iron requirement distributions are unknown. For women between 14 and 50 years, if users specify a binary variable in the 24-h recalls indicating the individual OC use, the SIMPLE macro will use the OC use-appropriate reference (**Supplemental table 2**). If users do not specify a binary variable of the OC use due to unknown OC use status, the SIMPLE macro will use the “mixed population” reference values to the whole study population (**Supplemental table 2**). Because iron requirements presented by IOM for the “mixed population” assumes 17% OC users and 83% non-OC users, estimation errors will likely occur if the study population has a higher or lower percentage of OC users than that of the reference population. Therefore, it is recommended that users provide a binary variable indicating individual OC use whenever possible.

1. **Men (male ≥ 9 y)**

Men’s iron requirement differs by age (9 - 13, 13 - 18, and ≥ 19 y). The SIMPLE macro applies the full probability method in estimating inadequate iron intake using age-appropriate reference values (**Supplemental table 3**).

Supplemental table 1: Probabilities of inadequate iron intakes and associated ranges of usual intake among children.

| Probability of Inadequacy | 6- 11.9 mos, mg/d | 1-3 y, mg/d | 4-8 y, mg/d |
| --- | --- | --- | --- |
| 1 | < 3.01 | < 1.0 | < 1.33 |
| 0.96 | 3.01 - 3.63 | 1.0 - 1.24 | 1.33 - 1.64 |
| 0.93 | 3.64 - 4.35 | 1.25 - 1.54 | 1.65 - 2.05 |
| 0.85 | 4.36 - 5.23 | 1.55 - 1.96 | 2.06 - 2.63 |
| 0.75 | 5.24 - 5.87 | 1.97 - 2.32 | 2.64 - 3.13 |
| 0.65 | 5.88 - 6.39 | 2.33 - 2.66 | 3.14 - 3.62 |
| 0.55 | 6.40 - 6.90 | 2.67 - 3.01 | 3.63 - 4.11 |
| 0.45 | 6.91 - 7.41 | 3.02 - 3.39 | 4.12 - 4.64 |
| 0.35 | 7.42 - 7.93 | 3.40 - 3.82 | 4.65 - 5.27 |
| 0.25 | 7.94 - 8.57 | 3.83 - 4.38 | 5.28 - 6.08 |
| 0.15 | 8.58 - 9.44 | 4.39 - 5.25 | 6.09 - 7.31 |
| 0.08 | 9.45 - 10.17 | 5.26 - 6.06 | 7.32 - 8.45 |
| 0.04 | 10.18 - 10.78 | 6.07 - 6.81 | 8.46 - 9.52 |
| 0 | > 10.78 | > 6.81 | > 9.52 |

^1^Table is adapted from the IOM tables I-5 (1). Iron absorption is assumed to be 18%.

Supplemental table 2: Probabilities and associated appropriate iron requirement values for non-pregnant and non-lactating women^1^

| Probability of Inadequacy | 9 - 13 y,  mg/d | 14-18 y | | | 19-50 y | | | ≥ 51 y,  mg/d |
| --- | --- | --- | --- | --- | --- | --- | --- | --- |
|  |  | Non-OC users,  mg/d | OC users,  mg/d | Mixed population,  mg/d | Non-OC users,  mg/d | OC users,  mg/d | Mixed Population,  mg/d |  |
| 1 | <3.24 | <4.63 | <4.11 | <4.49 | < 4.42 | <3.63 | <4.18 | <2.73 |
| 0.96 | 3.24 - 3.60 | 4.64 - 5.06 | 4.11 - 4.49 | 4.49 - 4.92 | 4.42 - 4.88 | 3.63 - 4.00 | 4.18 - 4.63 | 2.73 - 3.04 |
| 0.93 | 3.61 - 4.04 | 5.07 - 5.61 | 4.50 - 4.97 | 4.93 - 5.45 | 4.89 - 5.45 | 4.01 - 4.45 | 4.64 - 5.19 | 3.05 - 3.43 |
| 0.85 | 4.05 - 4.59 | 5.62 - 6.31 | 4.98 - 5.57 | 5.46 - 6.14 | 5.46 - 6.22 | 4.46 - 5.06 | 5.20 - 5.94 | 3.44 - 3.93 |
| 0.75 | 4.60 - 4.98 | 6.32 - 6.87 | 5.58 - 6.05 | 6.15 - 6.69 | 6.23 - 6.87 | 5.07 - 5.52 | 5.95 - 6.55 | 3.94 - 4.30 |
| 0.65 | 4.99 - 5.33 | 6.88 - 7.39 | 6.06 - 6.48 | 6.70 - 7.21 | 6.88 - 7.46 | 5.53 - 5.94 | 6.56 - 7.13 | 4.31 - 4.64 |
| 0.55 | 5.34 - 5.66 | 7.40 - 7.91 | 6.49 - 6.89 | 7.22 - 7.71 | 7.47 - 8.07 | 5.95 - 6.35 | 7.14 - 7.73 | 4.65 - 4.97 |
| 0.45 | 5.67 - 6.00 | 7.92 - 8.48 | 6.90 - 7.34 | 7.72 - 8.25 | 8.08 - 8.76 | 6.36 - 6.79 | 7.74 - 8.39 | 4.98 - 5.30 |
| 0.35 | 6.01 - 6.36 | 8.49 - 9.15 | 7.35 - 7.84 | 8.26 - 8.92 | 8.77 - 9.63 | 6.80 - 7.27 | 8.40 - 9.21 | 5.31 - 5.68 |
| 0.25 | 6.37 - 6.78 | 9.16 - 10.03 | 7.85 - 8.47 | 8.93 - 9.77 | 9.64 - 10.82 | 7.28 - 7.91 | 9.22 - 10.36 | 5.69 - 6.14 |
| 0.15 | 6.79 - 7.38 | 10.04 - 11.54 | 8.48 - 9.47 | 9.78 - 11.21 | 10.83 - 13.05 | 7.92 - 8.91 | 10.37 - 12.49 | 6.15 - 6.80 |
| 0.08 | 7.39 - 7.88 | 11.55 - 13.08 | 9.48 - 10.42 | 11.22 - 12.74 | 13.06 - 15.49 | 8.92 - 9.90 | 12.50 - 14.85 | 6.81 - 7.36 |
| 0.04 | 7.89 - 8.34 | 13.09 - 14.80 | 10.43 - 11.44 | 12.75 - 14.39 | 15.50 - 18.23 | 9.91 - 10.94 | 14.86 - 17.51 | 7.37 - 7.88 |
| 0 | >8.34 | >14.8 | >11.44 | > 14.39 | > 18.23 | >10.94 | >17.51 | > 7.88 |

^1^This table is adapted from the IOM tables I-6 and I-7 (1). Iron absorption is assumed to be 18%. Mixed population assume 17% of OC use in the population. OC, Oral Contraceptive.

Supplemental table 3: Probabilities of inadequate iron intakes and associated ranges of usual intake among men.

| Probability of inadequacy | 9 - 13 y,  mg/d | 14-18 y,  mg/d | ≥19 y,  mg/d |
| --- | --- | --- | --- |
| 1 | < 3.91 | < 5.06 | < 3.98 |
| 0.96 | 3.91 - 4.23 | 5.06 - 5.42 | 3.98 - 4.29 |
| 0.93 | 4.24 - 4.59 | 5.43 - 5.85 | 4.30 - 4.64 |
| 0.85 | 4.60 - 5.03 | 5.86 - 6.43 | 4.65 - 5.09 |
| 0.75 | 5.04 - 5.36 | 6.44 - 6.89 | 5.10 - 5.44 |
| 0.65 | 5.37 - 5.64 | 6.90 - 7.29 | 5.45 - 5.74 |
| 0.55 | 5.65 - 5.89 | 7.30 - 7.69 | 5.75 - 6.03 |
| 0.45 | 5.90 - 6.15 | 7.70 - 8.08 | 6.04 - 6.32 |
| 0.35 | 6.16 - 6.43 | 8.09 - 8.51 | 6.33 - 6.65 |
| 0.25 | 6.44 - 6.76 | 8.52 - 9.03 | 6.66 - 7.04 |
| 0.15 | 6.77 - 7.21 | 9.04 - 9.74 | 7.05 - 7.69 |
| 0.08 | 7.22 - 7.58 | 9.75 - 10.32 | 7.70 - 8.06 |
| 0.04 | 7.59 - 7.91 | 10.33 - 10.83 | 8.07 - 8.49 |
| 0 | > 7.91 | > 10.83 | > 8.49 |

^1^Table is adapted from the IOM tables I-6 and I-7 (1). Typographical errors from the original table were corrected. For example, for 14-18 years old, the iron intake range for the probability of inadequate intake of 0.55 should be 7.30-7.69 instead of 7.80-7.65 listed in the original table. Iron absorption is assumed to be 18%.

Supplemental figure 1: Population categories used to select appropriate reference values for iron intake among women^1^.


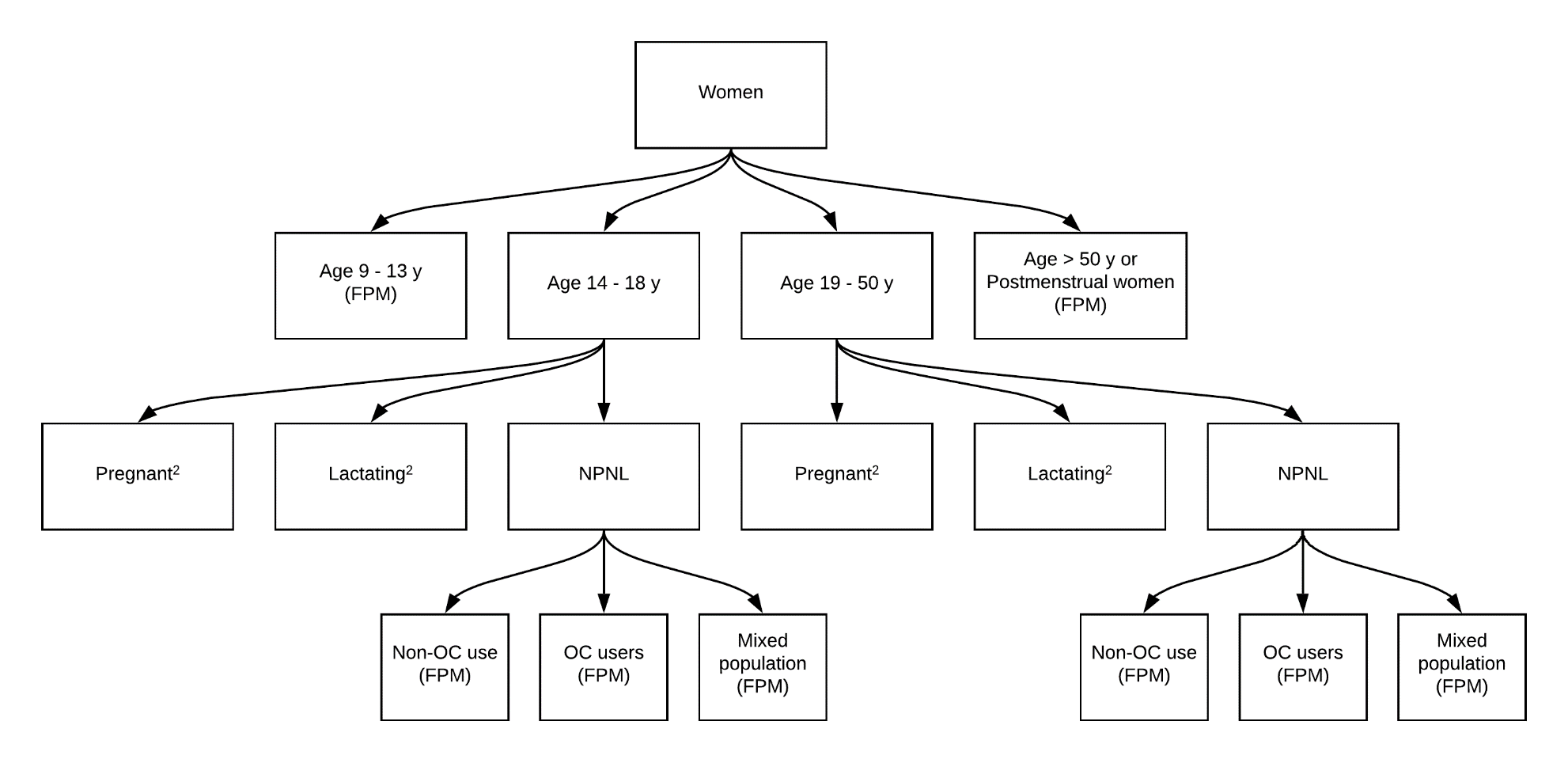


^1^FPM, Full probability Method; NPNL, non-pregnant or non-lactating women; OC, Oral Contraceptive.

^2^The distribution of iron requirement for pregnant or lactating women is unknown.

**Supplemental references:**

1. Food and Nutrition Board, Institute of Medicine. Iron. Dietary Reference Intakes for Vitamin A, Vitamin K, Arsenic, Boron, Chromium, Copper, Iodine, Iron, Manganese, Molybdenum, Nickel, Silicon, Vanadium, and Zinc. Washington, DC: National Academies Press (US); 2001.

2. Hurrell R, Egli I. Iron bioavailability and dietary reference values. The American Journal of Clinical Nutrition. American Society for Nutrition; 2010;91:1461S–1467S.

3. Armah SM, Carriquiry A, Sullivan D, Cook JD, Reddy MB. A complete diet-based algorithm for predicting nonheme iron absorption in adults. Journal of Nutrition. 2013;143:1136–40.

4. Tooze JA, Midthune D, Dodd KW, Freedman LS, Krebs-Smith SM, Subar AF, Guenther PM, Carroll RJ, Kipnis V. A new statistical method for estimating the usual intake of episodically consumed foods with application to their distribution. Journal of the American Dietetic Association. 2006;106:1575–87.

5. Food and Nutrition Board, Institute of Medicine. Using the estimated average requirement for nutrient assessment of groups. In: Institute of Medicine (IOM), editor. Dietary reference intakes: applications in dietary assessment. Washington, D.C.: National Academies Press (US); 2000.

6. Dietary Reference Intake, Institute of Medicine. Iron Intakes and Estimated Percentiles of the Distribution of Iron Requirements from the Continuing Survey of Food Intakes by Individuals (CSFII), 1994–1996. In: Institute of Medicine, editor. Dietary Reference Intakes for Vitamin A, Vitamin K, Arsenic, Boron, Chromium, Copper, Iodine, Iron, Manganese, Molybdenum, Nickel, Silicon, Vanadium, and Zinc. National Academy Press; 2001. pp. 701–3.

7. Armah SM, Carriquiry AL, Reddy MB. Total Iron Bioavailability from the US Diet Is Lower Than the Current Estimate. The Journal of nutrition. 2nd ed. 2015;145:2617–21.

8. Hurrell R, Egli I. Iron bioavailability and dietary reference values. The American Journal of Clinical Nutrition. 2010.

9. National Research Council US Subcommittee on Criteria for Dietary Evaluation. The Probability Approach. Nutrient Adequacy: Assessment Using Food Consumption Surveys. National Academies Press (US); 1986.

10. Australia Bureau of Statistics. Beaton's full probability method for iron [Internet]. National Academies Press (US); 2015 [cited 2016 Apr 30]. Available from: http://www.abs.gov.au/AUSSTATS/abs@.nsf/Lookup/4363.0.55.001Chapter6510312011-13

1. Postmenstrual women is defined as women who are > 50 years old. [↑](#footnote-ref-1)
